# The Profession and the Man

**Published:** 1896-01

**Authors:** Edwin H. Hughes

**Affiliations:** Newton Centre, Mass.


### THE PROFESSION AND THE MAN.[1]

BY REV. EDWIN H. HUGHES, NEWTON CENTRE, MASS.

Mr. President and Gentlemen,—A glance into a book a few years ago was rewarded by the interest of the following tradition, given now according to the memory of a hurried reading: There once dwelt in Hyderabad, India, a man whose name was Alhafed. One day there came to his fine country home a ministerial guest in the person of a Buddhist priest who gave to his host this crude account of the world's making: Back in the uncounted centuries, in the place of our revolving earth there was stationed a large, circular mass of thin vapor. In his own time the Almighty stretched forth his arm, placed his forefinger in the centre of this misty globe, and began to whirl it with infinite rapidity. The mighty circle became a flame and sped on in its fiery course. When the motion ceased the ball began to cool and contract. Mountains burst forth from its sides. The surrounding atmosphere rushed against it and

---

[1] An address delivered before the American Academy of Dental Science, at Young's Hotel, Boston, November 13, 1895.

depressed the surface, making beds for the oceans. According to the conditions of the cooling process rock was formed in one place, coal in another, and diamonds in a third. But Alhafed asked the priest what a diamond was. The stone was described and its value stated. "Where are diamonds found," was asked, with sparkling eyes. The answer was that the gems usually lay where a swift stream passed over white sand. Alhafed immediately became discontented. He sold his home and farm, collected his moneys, and started out in search of diamonds. He went from land to land, and after wanderings, long and vain, he stood at last upon the shores of Spain and gazed out over the waters of the Mediterranean. The sun shone upon the wave-crests and reminded him of the long-sought riches. Crazed with disappointment he cast himself into the sea, and his poor body was carried away through the rocks of Gibraltar. Meanwhile, the man to whom Alhafed had sold his farm toiled on industriously. One day as he worked in his garden he saw a shining stone in the sand of a little stream. He picked it up, carried it into his house, and placed it on a shelf. Shortly there came to call the same Buddhist priest. He spied the precious stone in its carbon case, and excitedly asked, "Where did you get this?" The man led him forth to the garden stream, and stooping down the priest drew forth diamond after diamond. Alhafed's successor became fabulously rich; for he was the owner of the famous Golconda mines.

The untrustworthy legend gives a trustworthy moral. Men are ever leaving the ordinary with a view to finding the extraordinary. They do not expect to find diamonds while engaged in the plain pursuit of gardening. And very often in their proneness to consider the best things as distant and exceptional, they fail to gain the treasures that lie near at hand. Nor does this mistake confine itself simply to matters commercial and material. Supposing the weird story as history rather than as allegory, it is strictly true that the luckless Alhafed would have found more character as well as more wealth if he had not been seized with discontent of his occupation. And this moral view is the one which has point for us. For while it is not likely that some men of to-day will leave an honest profession and go in search of Captain Kidd's treasure or the casket at the rainbow's foot, we may yet be captured by the thought that the manhood for which we long and strive is to be gained and deepened only apart from the chosen work of our everyday lives. It therefore comes about that our calling sinks to the level of immoral drudgery or never rises, at the highest, above a petty commercialism. It is possible for a man to lose himself, his brain, his heart, his very soul, in his profession. It has been told as a matter of joking that somewhere in the Old World there is a tombstone with this inscription: "John Jones. Born a man; died a grocer." But, ridiculous as the odd epitaph may seem, it really suggests the record of too many lives. It is not at all beyond sober truth to say that some, born as men and with the possibilities of high manhood, have died simply grocers or preachers or dentists. The intense specialism of our time has its advantages as pointed out to you by abler men; yet this same specialism has its dangers. If there be intelligent persons who contend that men are made by circumstances, we may, while being unwilling to allow the fulness of their claim, still grant that it will be hard to get a broad spirit out of a narrow work.

It is singular and anomalous, however, that in this age of rigid specializing there should still be the loud demand for breadth. It is hopeful that this demand is made not only upon belief, but also upon conduct. The time was when whole areas of life were put beyond the reach of morality. The edges of that dark period, sometimes even now, are seen amid the light of the present wider conscience. One—Mackenzie Wallace—says that in Russia such incidents as the following are still possible: A house-breaker, when in the act of robbing a church, finds it hard to extract the jewels from an image; he thereupon makes a vow that if a certain saint will assist him he will place a ruble's worth of candles before the saint's statue. A peasant prepared to rob a young man connected with the Austrian embassy in St. Petersburg. At length he kills his victim; but before doing so he enters a church and commends his bloody undertaking to the divine protection. A robber murders and rifles a traveller, but refuses to eat a piece of cooked meat, which he finds in the cart, because, indeed, it happened to be a fast-day. Extreme cases such as these will illustrate the efforts that have been made to mingle light and darkness and yet live in both. It was a distinct advance towards righteousness when it became the general verdict that a man's morality was the very essence of vanity and pretence, unless it kept him from outbreaking crimes. But our broadened thought now recognizes that this rule does not go far enough. It is at best simply negative. So there is at last heard a burning demand that the cheap and false distinction between things sacred and things secular shall be utterly wiped out; that men shall so use their constant work as to make it a means and expression of character; that the heart shall ever command obedience from the head and hand, and that no longer professionalism shall devour manhood. It is sure that this lofty and heroic ideal demands a large and serious purpose. No superficial person will reach it. If there is anything so sad as to see a man who could be big in intention and in heart, toying with some little work or movement, it is to see a little man taking hold of some great profession with a puny purpose. We need to come to our regular occupations with a thoughtful and humane spirit. There is too much tendency to regard our work as a grim necessity foisted upon life, as a punishing curse, as a rank intruder. But such a view needs to be banished. The thoughtful man will find enough of moral bearing and beauty in his calling to lift it out of the degradation of drudgery or commercialism, and to set it far on high.

There will be, first of all, the knowledge that his work stands for a genuine need of human life. It is a fact that sometimes we grow weary of this claim, especially when it comes from the mouths of men whom we suspect not to use it sincerely. The simpering agent who is out for the bread and butter purpose, who yet seeks to invest his work with the purely benevolent and missionary air, often puts a heavy task upon our patience. But if hypocrisy be the tribute which vice pays to virtue, the protests of the insincere represent the attitude towards their employment that true men should seek to hold. For in truth every calling or profession stands for some necessity in life. There is no trade so humble as not to gain dignity from this view. The carpenter may say, It is absolutely necessary that people should have homes in which to dwell, wagons in which to travel, boats in which to sail, cars in which to ride, churches in which to worship. Viewing his work from the stand-point of its essential relation to life, the carpenter may rightfully claim that his occupation be somewhat exalted. The merchant may say, It is positively needful that people should be provided with eatables, with coal, with oil, with warm garments, with household conveniences. The mercantile calling represents, therefore, a plain and imperative need of the world, and should be held in high respect. The lawyer may say, The relations of men are not yet perfect. The organized life of society creates questions and crises. Rogues are alive and busy. Honest men are sometimes obstinate and unreasonable. The troubled world needs legal advice. Having such responsible duties, the legal profession should be given high regard. The doctor, whether of medicine or of dental surgery and science, may likewise make an impressive plea. He may say, There are many ills that flesh is heir to. People are suffering. Subtile disease is floating in the atmosphere. Both the prevention and the cure of aches and pains are constantly needed. The people must rely upon a quick eye, a knowing mind, a trained hand in order that their afflictions be relieved. Having for its object the preservation of health and the alleviation of physical woes, the medical profession should be assigned a high value. Now, all these claims are just and should be readily granted. He who holds in contempt any vital employment of life and casts discredit upon its followers has a false idea and is in sore need of moral enlightenment. It is not contended that man in the midst of the work which supplies his personal wants should be forever posing with the air of a philanthropist. That would scarcely be an honest attitude. The urgent needs that press upon each one of us, the needs of shelter, clothing, food, will afford a large and proper motive for professional activity. But it is insisted that a man should view his work as it relates to other men, and not simply as it relates to himself. And he who in selfish greed applies his powers to his daily tasks, and while grasping gold in payment never considers that through the years he has been giving a good, safe contribution to life; that he has been sheltering bodies, satisfying hunger, defending the troubled, or relieving the suffering; he who fails to get this view of his tasks has all the while been becoming more of a professionalist and less of a man. The person who does not seek the utmost skill and buy the best instruments for his dental work, not only because he wishes to get larger prices, but also because he wishes to render better service to his customers, and to give an honest response to a need of human life, is most certainly sinking his manhood in his dentistry. It is surely not too much to ask that men come to their regular vocation in this generous spirit. The great poetess of England has some lines in which she teaches that the largeness of one's work will be determined by the individual purpose. Her statement is that it is better to be a tight-rope walker with a hearty thought than to be a poet with a superficial aim:

> "I would rather dance
> At fairs on tight rope, till the babies dropped
> Their gingerbread for joy, than shift the types
> For tolerable verse, intolerable
> To men who act and suffer. Better far
> Pursue a frivolous trade by serious means
> Than a sublime art frivolously."

If, therefore, a man has an employment which is not frivolous, an employment which makes an essential part of our great and complex life, he needs to face it with moral pride and earnestness.

There is, moreover, a thought which will give a man's work height and aspiration just as this already stated will give it breadth and sympathy. Any true profession or work has a divine side. There is a large suggestiveness in certain portions of the religious history which we call the Bible,—suggestiveness for this particular point. Frequently it is represented that the call to the very highest life and leadership came to men as they were engaged in their ordinary pursuits. The call to the most majestic position as general, legislator, and ruler that ever came to man was received by one who was quietly tending his flocks upon the mountain-side. The first king of a mighty nation went out on a faithful search for his father's herds, and instead he found a kingdom. The shepherds who kept the quiet watch over the sheep on the Judean plains were the men who heard the thrilling advent song and gained the honor of the first worship. Matthew was busy at the table of the tax-gathers when he was summoned to the discipleship that gave him immortal glory. John and James were engaged in the common occupation of fisherman when they heard the voice of authority, and pulled their boat over the blue waves to come ever nearer and nearer to Him who was to dominate the future. These incidental touches are suggestive. They mean, at the least, that the daily employment does not, need not, conceal the highest things from the worker. For it is not over-bold to say that to the reverent eye every vocation reveals things and powers which came from a divine agency. The carpenter handles wood which a power, not himself, has been a hundred years in making. The painter mixes colors which some power has driven across the ninety-two million miles from the sun and stored in metal and in planet. The drivers on our street-cars grasp the unseen force upon the upper and lower wires, and an invisible hand pushes the loads of busy men through the streets. There is no man whose work does not open up to him infinite distances, and who may not catch divine messages in the midst of his occupation. It was the thought of old Stradivarius, the violin-maker, that since a divine power had put the strange harmonies in the strings and cavities, he who so combined the conditions as to make the best instrument was in reality nothing less than a partner with the Infinite. It is no wonder that George Eliot, in her "Stradivarius," should exalt the man's dignified thought of his work. She represents the faithful artist as saying,—

"Who draws a line and satisfies his soul,
Making it crooked where it should be straight?
An idiot with an oyster-shell may draw
His lines upon the sand all wavering,
Fixing no point or pathway to a point;
An idiot one remove may choose his line,
Straggle and be content; but, God be praised,
Antonio Stradivari has an eye
That winces at false work and loves the true,
With hand and arm that play upon the tool
As willingly as any singing bird
Sets him to sing his morning roundelay,
Because he likes to sing and likes the song."

But his friend Naldo says,—

"'Tis a pretty kind of fame
At best that comes of making violins;
And saves no masses either. Thou wilt go
To purgatory none the less."

But Stradivarius replies,—

"'Twere purgatory here to make them ill;
And for my fame—when any master holds
'Twixt chin and hand a violin of mine,
He will be glad that Stradivari lived,
Made violins, and made them of the best.
The masters only know whose work is good;
They will choose mine; and while God gives them skill,
I give them instruments to play upon,
God choosing me to help Him."

What reason is there to prevent every honest man who loves his profession, who thinks of it as responding to human needs, from having this noble thought of his work? When once we rise to such a view we are sure to lift our employments with us above either wearying drudgery or ambitious greed.

And as every profession has a human breadth and a divine height, so also does it have a personal point. By this it is not meant that a profession provides a man with food and shelter and other needed good. This it ought to do, and does do. But the meaning is that every man's employment relates itself vitally to the man's character. It is not at all the intention to engage now in any subtile and refined moral psychology. We will leave that task to the schools. Men, however, are far too likely to have a cheap thought of the influence which their professional activities shall have upon themselves. It would be a sad and disastrous view if we were driven to conclude that the things to which a man gives three-fourths or two-thirds of his conscious life were wholly immoral, so much so that the doing of them would in no real way contribute to his higher being. For many decades, Gladstone, the prime-minister of England, has engaged each week-day in chopping wood in the Hawarden forest. He has done this, not with the idea of supplying fire-wood for the castle, but rather with the idea of supplying himself with muscular power, so that he might meet the pressing demands of statesmanship. Now, it is simply incredible to think that the greatest statesman of the British empire receives a physical reflex influence from his work as a wood-chopper, and yet does not receive a mental and moral reflex influence from his profession as an official and reformer. There is a scientific doctrine of the persistence of force. And there is a sure doctrine of moral persistence. No man escapes from his own work. His profession refuses to be shaken off. It haunts him though unseen, dogs him though invisible, sleeps with him in the darkness, and comes back to the next day's work to toil with him again. Yet this is not the ordinary view. At the end of a day's labor one man drops his hammer and nail-pouch and says, "Done." It is not true. The day's work is only begun. Another man drops his yard-stick and says, "Done." It is not true. The day's work is only begun. Another man puts down his forceps or mallet, stops his whirling wheel, dismisses his patient, and says, "It is done." But if it be true that the physical skill gained from the day's task is to reside henceforth in the man's arms and hands, it is safe to say that the moral influence of that work is to remain ever in the man's soul. There is a true and deep sense in which every man's employment stays with him perpetually. The work that we do on all lines will insist on continued association with our souls. And this view of one's occupation will redeem from wrong views as to failure. We may see the house which represents ten years of a man's work and saving go up in smoke and flame. We may cry out, "It is too bad! All the man's effort has been in vain." But that is a superficial view. It deals only with man as an animal. It allows no height, no breathing space. The most essential part of the ten years' work is in the man, in the patience, industry, honesty, keenness, love, that have day by day rebounded to his soul. Certainly this idea deepens one's thoughts of his profession, and delivers it forever from a cheap and temporary place in his life.

It must surely be, then, that these three conceptions of employment will set our daily work on high. To the man who comes to regard his profession as responding to human need, as fitting itself to divine co-operation, and as pushing its influence backward forever on his own soul, that profession will forever contribute to manhood, and will forever call the growing manhood to its service. It will thus be seen that the tribute which Mrs. Browning in her Aurora Leigh pays to the world's moral teachers is no piece of poetic extravagance:

"I write so
Of the only truth-tellers now left to God,
The only speakers of essential truth,
Opposed to relative, comparative,
And temporal truths; the only holders by
His sun-skirts, through conventional gray-glooms;
The only teachers who instruct mankind,
From just a shadow on a charnel-wall,
To find man's veritable status out
Erect, sublime,—the measure of a man;
And that's the measure of an angel, says
The apostle. Ay, and while your common men
Lay telegraphs, gauge railroads, reign, reap, dine,
And dust the flaunty carpets of the world
For kings to walk on, or our president,
The poet suddenly will catch them up
With his voice like a thunder,
'This is soul!
This is life, this word is being said in heaven.
Here's God down on us! what are you about?
How all those workers start amid their work
Look round, look up, and feel, a moment's space,
That carpet dusting, though a pretty trade,
Is not the imperative labor after all.'"

